# The Value of Vaccines: A Tale of Two Parts

**DOI:** 10.3390/vaccines10122057

**Published:** 2022-11-30

**Authors:** Nathan Fox, Philip Adams, David Grainger, Jennifer Herz, Carolyn Austin

**Affiliations:** 1 Biointelect, Sydney 2000, Australia; 2 Victoria University Centre of Policy Studies, Melbourne 3000, Australia

**Keywords:** COVID-19, economic evaluation, health technology assessment, vaccines, value

## Abstract

Vaccines are essential to ensuring a nation’s health, wellbeing and prosperity. After the coronavirus pandemic commenced, the Australian Government introduced social restrictions to constrain virus transmission, seeing significant economic impacts. Reflecting the extraordinary circumstances, subsequent vaccination rollout forwent usual health technology assessment (HTA) processes, facilitating restrictions removal and leading to societal and economic recovery. However, in ‘usual’ circumstances, HTA may not consider such broader effects of vaccines, making it challenging for them to achieve timely funding. We used detailed modelling to compare economic impacts under continued lockdowns against population-wide vaccination rollout between January 2020 and June 2023 and examined global HTA vaccine evaluation methodologies and efforts to develop broader valuation approaches. Australian gross domestic product reduces by approximately AUD 395 billion with lockdowns. With vaccination rollout, this effect is approximately AUD 214bn, a positive incremental impact of AUD 181bn. Vaccination contributes to large estimated positive effects for tourism (AUD 28bn) and education (AUD 26bn) exports, employment (142,000 jobs) and government finances (AUD 259bn). Conversely, global HTA methods generally only consider direct patient health outcomes and healthcare system-related costs, with broader effects usually not impacting funding decisions. Our results suggest that recent efforts to propose broader HTA valuation frameworks warrant further policy consideration.

## 1. Introduction

Since the mid-20th century, vaccination has been an essential component of national strategies to eradicate or reduce the impact of infectious diseases [1]. After the Severe Acute Respiratory Syndrome (SARS) coronavirus (COVID-19) pandemic commenced in December 2019, the Australian Commonwealth Government, along with state and territory governments, employed comprehensive social restrictions to contain disease spread and minimise healthcare system pressures [2]. As of November 2022, the pandemic is estimated to have resulted in 642 million confirmed cases and 6.63 million deaths [3]; in Australia, this has been estimated to be 10.7 million cases and 16,119 deaths (i.e., approximately 0.15% of all cases) [4]. The pandemic has led not only to significant adverse health outcomes and healthcare system strains, but global contraction in economic activity, with Australia experiencing its first recession since 1991 [5]. This subsequently led Australia’s Government to enact large-scale fiscal policy measures to support its economy and healthcare system [6].

Subsequently, like other countries, Australia’s Government sought to rapidly acquire and distribute vaccines, bypassing established health technology assessment (HTA) procurement processes [7] and using Advanced Purchase Agreements to secure vaccine supply [8,9]. HTA is a formal process that uses explicit methods to determine the value of a health technology, with considerations of value ‘often including clinical effectiveness, safety, costs and economic implications, ethical, social, cultural and legal issues, organizational and environmental aspects, as well as wider implications for the patient, relatives, caregivers, and the population’ [10]. Over time, it has become increasingly used by independent agencies and payers such as governments to inform, recommend or make funding decisions by evaluating health intervention (including vaccines) ‘value for money’ relative to existing medical management approaches. HTA is well established in Australia [11], the United Kingdom (UK) [12], Europe [13], Canada [14], some parts of the United States healthcare system [15], and increasingly, in Asia [16].

In Australia, vaccines reimbursement follows a lengthy, multi-stepped process. After initial clinical consideration by the Australian Technical Advisory Group on Immunisation [17], the Pharmaceutical Benefits Advisory Committee (PBAC), an independent expert advisory body, undertakes formal HTA [18]. A positive PBAC recommendation, with cost-effectiveness a key quantitative PBAC decision making criteria [19], followed by Commonwealth Government approval, is required before a vaccine can be listed on the National Immunisation Program [20] for subsidised use by eligible patients. As of November 2022, the PBAC has not considered COVID-19 vaccination funding submissions. However, it has considered COVID-19 therapies for mild-to-moderate patients at risk of hospitalisation, as well as pre-exposure prophylaxis for individuals who are severely immunocompromised or for whom available funded vaccinations were contraindicated [21,22,23]. In the UK, HTA advice processes have been used to inform COVID-19 vaccine coverage recommendations [24], albeit without inclusion of cost-effectiveness analysis (in contrast to prior vaccine funding decisions [25]). As the world moves from pandemic to pandemic recovery, it remains to be seen when and how COVID-19 vaccines will be evaluated as a manner of course through established HTA processes.

**Figure 1:**
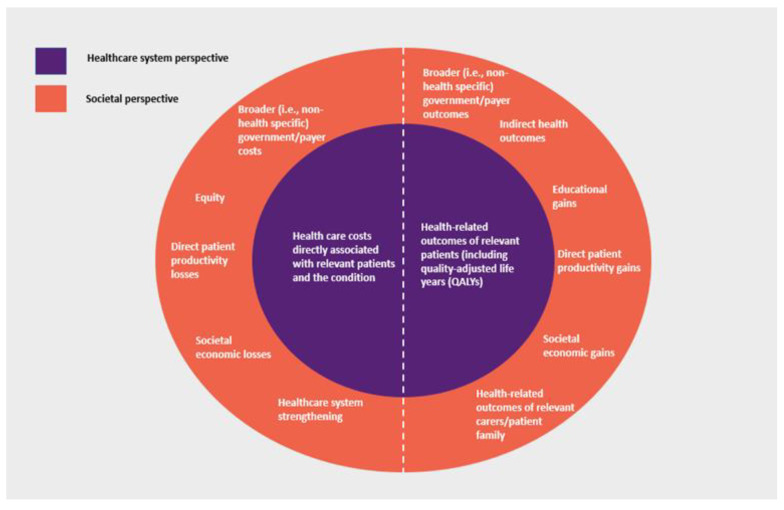
HTA economic evaluation: healthcare system perspective versus societal perspective. Notes: The societal perspective also includes costs and outcomes considered by the healthcare system perspective. The selection of societal perspective considerations is not intended to be exhaustive. Abbreviations: ‘QALYs’, quality-adjusted life years.

A key methodological consideration of HTA is defining the evaluation ‘perspective’, i.e., the scope of costs and outcomes considered. While there may be practical variations, HTA generally takes one of two approaches, reflecting where costs and outcomes occur and whether they are directly or indirectly attributable to the condition and medical intervention being considered. The narrower ‘healthcare system perspective’ generally includes health care costs (including those incurred by the patient) and health-related outcomes directly attributable to the treatment, disease and patient being considered. In many countries, this perspective reflects the government role as ‘payer’ for health care (i.e., a ‘payer perspective’). In addition to these, the broader ‘societal perspective’ may also consider additional costs and outcomes that impact patients, family and carers of patients, governments and payers, and society overall, with potentially various elements of value being able to be considered [26] (Figure 1).

HTA methods in many countries, including Australia, generally only consider direct patient health outcomes and healthcare system-related costs directly associated with the patient and condition, with broader impacts on society not necessarily included in ‘base case’ analysis [27,28]. Therefore, depending on the relative distribution or ‘location’ of costs and outcomes for a particular condition and treatment, this may affect HTA economic evaluation outcomes and ultimately, societal resource allocation decisions.

This is especially relevant for vaccines. Infectious diseases, including COVID-19, have unique characteristics that must be considered when valuing vaccine interventions: disease transmissibility, evolution in their nature over time, large-scale population health outcome impacts and as COVID-19 has demonstrated, the risk of widespread societal effects, including those which are felt beyond the immediate patient. Similarly, the positive effects of vaccines may be felt beyond the scope of health and the healthcare system and be long term in nature.

We believe, therefore, that as a HTA methodological consideration, an appropriate perspective is critical to ensuring the conferral of the broad range of positive effects and outcomes of vaccines to society. With the COVID-19 pandemic, it is timely to consider the broader economic and societal impacts of vaccines and the importance of considering these in HTA. This study estimates the economic impacts of the COVID-19 pandemic and the subsequent mitigating effects of population-wide COVID vaccination in Australia. Current HTA approaches for assessing the value of vaccines (both for COVID-19 vaccines and more generally), along with recent efforts to consider broader HTA valuation frameworks to capture the true value of medical interventions, are then considered. In doing so, we demonstrate the value of vaccines extends beyond the traditional dimensions of direct health and healthcare system impacts, that current HTA value frameworks are too limited and that they should be expanded to capture the true broader societal value of vaccines.

## 2. Materials and Methods

### 2.1. Economic Modelling

We used the Victoria University Regional Model (VURM) [29], a Computable General Equilibrium (CGE) model, to estimate the comparative economic effects of maintaining and removing (following population-wide vaccination rollout) Australian government (Commonwealth, and State and Territory) social distancing restrictions, including border closures (‘shocks’), across the period January 2020 to June 2023.

CGE modelling is an established macroeconomic technique commonly used by government agencies to inform policy discussions and decisions [30]. It uses actual economic data to estimate how economic activity might react to changes in policy, technology, or other external factors (‘shocks’). The modelling aims to show the difference between two alternative future economic states, i.e., ‘with’ and ‘without’ the proposed change, by considering interactions between sections of the economy [31]. The VURM has been used extensively by government and private decision makers to evaluate the economic costs and benefits of policy changes and other external factors (such as pandemics) on the Australian economy. Recently, it has been used to evaluate the pandemic’s impact on Australia’s resource industries and work force needs. These reports among others can be found at: www.copsmodels.com/elecpapr.htm [32] (accessed on 10 October 2022).

The VURM operates on the principles of bottom-up aggregation of economic activity across all Australian geographical regions. Economic activity, as represented by the national account identity (i.e., the sum of consumption, investment, government spending, exports and imports (C+I+G+X-M)) is estimated for each region. Following neoclassical economic assumptions, markets are assumed to clear and to be competitive. ‘Agents’, or participants, of the economy are assumed to operate according to assumptions of cost-minimisation and utility maximisation [33].

Resulting analysis presents differences in key economic indicators across geographical regions and industry sectors. Using Australian and international economic data, the VURM estimates the impacts of these shocks on economic activity through 7 mechanisms: direct productivity impacts; demand; world economic effects; government fiscal policy; net overseas and interstate migration; direct, COVID-patient specific labour productivity impacts and COVID-related healthcare system expenditures.

The model assessed three scenarios: 1. No COVID: Where COVID-19 does not exist, and economic activity is not impacted. 2. Without vaccination: Governments maintain social restrictions. 3. With vaccination: Restrictions and economic outcomes experienced under Without vaccination until January 2021, when vaccination rollout commences, with required population levels to remove all restrictions across Australia reached by late 2021.

Acknowledging the complex and dynamic nature of the COVID-19 virus, simplifying assumptions were made: reflecting actual vaccine rollout [34] and the Commonwealth Government’s National Plan [35], Australia as a whole moves beyond 80% vaccination coverage (ages above 16 years) by the 4th quarter of 2021 and maintains coverage through booster doses; as per the Commonwealth Government’s National Plan, the majority of restrictions, including border closures, are subsequently removed Australia-wide [35]; the prevailing Australian COVID-19 variant upon restriction removal is the Delta variant [36].

Key macroeconomic outcomes, including for Australian gross domestic product (GDP), movement sensitive industries (tourism and education), national headcount employment and Commonwealth Government budgetary flows, were estimated. Two sensitivity analyses (‘upper’ and ‘lower’) estimating the effects of plausible halving and doubling of COVID burden (i.e., burden and/or severity) post-restriction removal impacting productivity and consumer demand in movement-sensitive sectors were conducted. Results are reported as percentage and absolute (in Australian dollars, 2021 price levels, undiscounted) deviations from the no COVID scenario. The model is solved with the General Equilibrium Modelling PACKage (GEMPACK) economic modelling software [37]. For further details on the operation and assumptions used in modelling, refer to Appendix A.

### 2.2. Literature Search

We conducted a targeted international review of current operational vaccine HTA methodologies of ten HTA agencies. We focused on countries with well-established, internationally acknowledged HTA disciplines and practices that have an influential role advising on vaccine reimbursement and funding, including Australia’s PBAC. In particular, HTA methodological guidelines were searched for and reviewed to confirm presence of guidance on HTA perspective, the guidance provided and its effect (e.g., whether compulsory or recommended).

Additional pragmatic literature searches for post-pandemic HTA-based COVID-19 vaccine evaluation studies, efforts to reform HTA valuation frameworks and options proposed to enhance HTA vaccine valuation scope were also conducted. Both published and grey literature sources were considered. A search of the databases PubMed and Scopus was conducted originally in late-October 2021, with subsequent search again in March to May 2022. HTA studies were reviewed and included in consideration if they were published in English and were perceived to attempt to conduct a health economic analysis or cost-effectiveness evaluation of COVID-19 vaccines, from a healthcare system perspective and/or a broader societal perspective. Studies that used both real clinical data from COVID-19 vaccines, or those that used theoretical or constructed pandemic and vaccination strategy scenarios, including estimates of key modelling and input assumptions, were considered. Similarly, works that considered the question of HTA valuation scope beyond the current traditional healthcare system perspective were reviewed and considered. The objective of these additional literature searches was to identify selected examples of works exploring HTA perspective and valuation framework considerations beyond current scope (Table 1) (Appendix C).

## 3. Results

### 3.1. Economic Impacts

In this section, we concentrate on VURM projections for the scenarios outlined above for 4 key variables: national real GDP (a measure of production), national employment, industry production and fiscal revenues. The simulation results are reported in figures and tables. The simulation results are driven by the exogenously imposed shocks explained in Appendix A.3 of Appendix A. As previously discussed in Methods, vaccination rollout in the With vaccination scenario commences from quarter 1, 2021.

#### 3.1.1. Gross Domestic Product (GDP)

**Figure 2:**
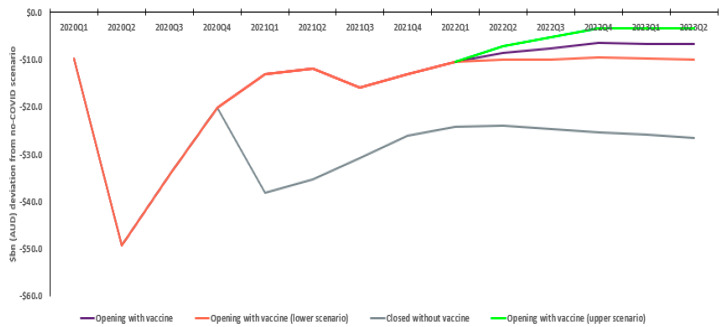
Real National GDP: Estimated deviation from no COVID scenario (AUD bn).

Without vaccination, Australian GDP is estimated to decline approximately AUD 395.2bn relative to the no COVID scenario. In the with vaccination scenario, reductions in the pandemic’s impact on the Australian economy steadily increase, peaking in quarter two 2021, with a total relative GDP decline of AUD 214.3bn. This sees an estimated positive economic difference of AUD 180.9bn, with the quarterly incremental economic outcome steadying at AUD 20bn by 2023. Sensitivity analysis shows this is not materially affected by differences in COVID severity post-restriction removal, ranging from AUD 167.7 to AUD 194.9bn (Figure 2).

#### 3.1.2. Education Exports

**Figure 3:**
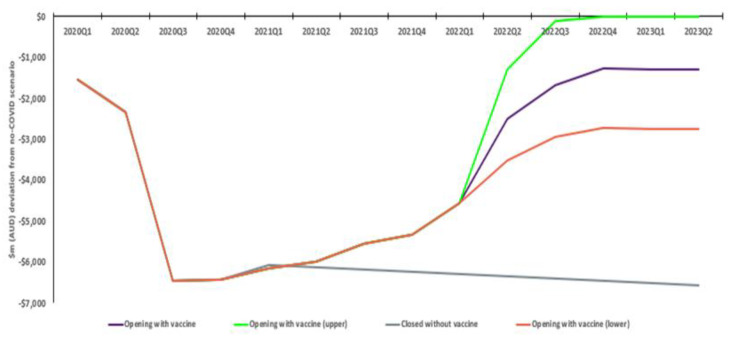
Real education exports: Estimated deviation from no COVID scenario (AUD bn).

Education exports initially drop approximately 82% relative to a no COVID scenario. However, there is a gradually increasing rate of recovery, with exports in the with vaccination scenario settling 15% below the no COVID scenario. Without vaccination, exports remain approximately 76% down, meaning an estimated incremental AUD 27.6bn economic benefit with vaccination. A more conservative response by international students to restriction removal sees an ultimate 32% reduction in activity relative to no COVID (approximate AUD 21.0bn benefit), while convergence of student exports to the no COVID scenario sees an estimated AUD 34.2bn benefit (Figure 3).

#### 3.1.3. Tourism Exports

**Figure 4:**
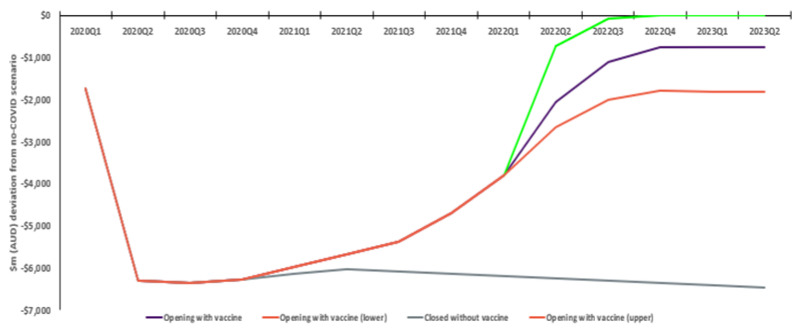
Real tourism exports: Estimated deviation relative to no COVID scenario (AUD bn).

Similarly, tourism exports initially drop approximately 92% without vaccination. With vaccination, there is a steady, then accelerating response, ultimately seeing activity 10% below the no COVID scenario. In contrast, without vaccination, exports stay 85% lower. The positive incremental economic impact of COVID vaccines is approximately AUD 25.7bn. A more conservative response by tourists sees an ultimate 24% reduction relative to no COVID (total incremental benefit AUD 22.1bn), while convergence to the ‘no COVID’ scenario sees an estimated total incremental AUD 29.6bn improvement (Figure 4).

#### 3.1.4. Employment

**Figure 5:**
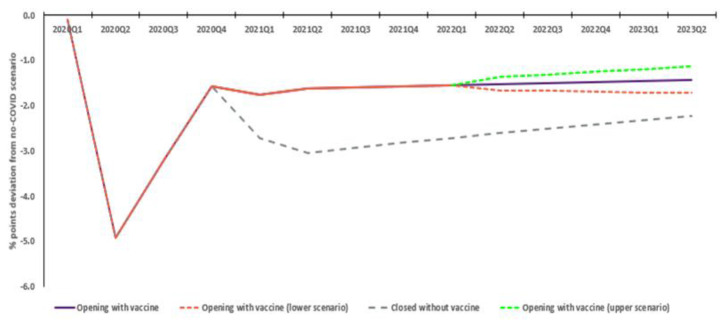
Employment: Estimated deviation national headcount employment relative to no COVID scenario (%). Note: Modelling includes actual Australian employment statistics up until quarter four 2021.

Initially, national employment headcount declined by approximately 640,000 jobs, before Commonwealth Government fiscal stimulus and other support measures saw an improvement. From quarter one of 2021, the with vaccination scenario sees the employment gap fall relative to no COVID. Without vaccination, the employment gap COVID rises and, while employment eventually recovers, remains significantly well below that with vaccination, with an average quarterly employment disparity between the two scenarios over the period of approximately 142,000 jobs (Figure 5).

#### 3.1.5. Commonwealth Government Budget

The estimated lockdown-related reduction in economic activity sees a sharp drop in Commonwealth Government revenues, while simultaneously seeing greater expenditures supporting pandemic response, the economy and society. The with vaccination scenario sees an estimated net positive AUD 258.6bn change in finances (i.e., revenues less expenditures) (Table 2). This illustrates improved taxation revenues and reduced expenditures due to improved health outcomes and economic activity. This table was populated using data from simulation of the VURM. The model-determined output was produced, in the main, from user-determined input. The input includes detailed information on Commonwealth and state and territory government COVID support payments and other related expenditure through schemes such as JobKeeper. More detail of support measures included is given in www.treasury.gov.au/coronavirus (accessed on 10 October 2022) [38]. For further details of the results of modelling, refer to Appendix B.

#### 3.1.6. Interpretation of Outcomes

A key question resulting from analysis is comparison of projections with resulting outcomes. At the time of running the simulations, statistics through to and including the second quarter of 2021 were available for a range of key economic variables from the Australian Bureau of Statistics (ABS). These cover national accounts, balance of payments and labour force statistics, and have been incorporated into our scenarios. Thus, for the with vaccination scenario, the charts and tables show observed outcomes to quarter 2 of 2021 and model-determined best-forecast projections thereafter.

The gap between the with vaccination and without vaccination scenarios yields hypothetical impacts of vaccine rollout. Unfortunately, without observed statistics on the impacts of vaccination alone prior to quarter 2 of 2021 it is impossible to provide statistical evidence on how close these projections are to reality. However, every effort has been made to include as many exogenous input changes in population, productivity, etc., that are clearly related to the pandemic, in modelling (see Appendix A.3 of Appendix A).

Nonetheless, the positive contribution the opening of borders domestic and international has had on Australian GDP outcomes across 2021–2022 has been clearly evident, with the ABS noting in its review of June 2022 quarter GDP, ‘The continued growth was aided by the first full quarter of re-opened domestic and international borders since the pandemic began.’ [39].

### 3.2. HTA Literature Review

#### 3.2.1. Current International HTA Evaluation Framework Perspectives

The current HTA practice and guidelines of ten leading HTA practicing countries (Australia, Canada, the UK, France, Germany, the Netherlands, Sweden, Belgium, New Zealand, and the United States (UK)) were considered. Although HTA practice has increasingly spread worldwide, these countries and HTA systems were selected on the basis of their long-established histories of HTA both as a discipline and in their practical use of HTA to inform funding decisions, as well as generally being those HTA systems referred to globally most commonly. Review was limited to ten for convenience and is not considered to influence findings unduly.

For these selected countries, review included consideration of ‘central’ HTA agencies (whether part of government departments, or independent/at arms’ length), as well as National Immunisation Technical Advisory Groups (NITAGs), which may provide immunisation advice to governments or HTA bodies [40]. Australia [17], the UK, Germany, France, Belgium, Canada, the Netherlands, Sweden, and the United States make public vaccination coverage decisions or recommendations through NITAGs [41,42,43,44,45,46,47,48]; all except Belgium’s providing for economic evaluation (in Belgium the KCE may conduct economic evaluations) (Table 3) [49]. Australia’s NITAG, ATAGI, provides its advice to the PBAC, who undertakes HTA required for ultimate funding and listing decisions [20].

Where economic evaluation is used, the healthcare system perspective is common, although there are examples of the societal perspective. Following the perspective of the National Institute for Health and Care Excellence (NICE) [41], which takes the healthcare system perspective with accounting for personal social services costs [50], the JCVI has had discretion to incorporate additional value elements in analyses [25]. Germany’s STIKO considers productivity impacts on patients and carers and direct non-medical costs [42]. Sweden’s Public Health Agency (FoHM) provides for a broad array of healthcare and societal value elements, including productivity losses of patients and caregivers [51]. Australia’s PBAC incorporates productivity losses and impacts on family and carers in sensitivity analyses only [28]. New Zealand’s Pharmaceutical Management Agency (PHARMAC) applies the same healthcare system perspective to vaccines as it does to other medicines and does not appear to allow societal perspective sensitivity analyses [52].

The Centre for Disease Control and Prevention’s Advisory Committee on Immunization Practices (ACIP) does not prescribe perspective, but provides for multiple perspectives, including that broader than a healthcare system perspective, i.e., provision for both the payer and societal perspective. In particular it notes, ‘The societal perspective typically includes all relevant medical costs regardless of payer, time costs of patients in seeking and receiving care, time costs of informal caregivers, transportation costs, effects on future productivity and consumption, and other effects occurring outside the healthcare sector.’ [53]. France’s Haute Autorité de santé (HAS) provides for a collective perspective, whereby all individuals or institutions affected in terms of healthcare system costs or benefits are considered. It notes, ‘Under a collective perspective, all resources consumed in the production of the overall patient care are taken into consideration. They cover the domestic sphere (e.g., informal care), the healthcare sphere (e.g., stays, procedures, and health products) and the medico-social sphere (e.g., stays, personal care services). Under the healthcare system perspective, the resources considered are those involved in the production of care (stays, procedures, and healthcare products)’. If the collective perspective is not used, a healthcare system perspective should be adopted [54]. In Canada, the National Advisory Committee on Immunization’s (NACI) *Guidelines for reporting model-based economic evaluations of vaccination programs in Canada* guidance on perspective notes, ‘publicly funded health system and societal at minimum, with societal considerations inclusive of, ‘productivity losses, consumption, and costs and outcomes of non-health sectors.’ [45]. It is noted that while some HTA guidelines have undergone review since the pandemic (e.g., in Canada and the UK), these were planned and scoped prior to the pandemic [45,55].

In summary, there is provision in some countries for a broader evaluation perspective, although the effects allowed in consideration are typically limited to those impacting the direct patient. Positively however, there is some provision for consideration of patient productivity impacts (e.g., in Canada, Germany, Sweden and the US). In some cases, they are only provisioned for in sensitivity analyses (e.g., Australia, France). In some cases (e.g., Australia), it remains unclear to what extent the HTA agency is obligated to consider these in its HTA decision making role [28].

#### 3.2.2. International and Australian HTA Funding Decisions

Historical HTA decisions indicate the impact of perspective on vaccine funding decisions. In 2013, the UK’s Joint Committee on Vaccination and Immunisation (JCVI) was requested to make a recommendation on meningococcal B immunisation programs [56]. It subsequently released an interim statement that even if the list price were GBP 0, the vaccine would not be cost effective [25]. Additional parameters not routinely included in analysis were subsequently incorporated by the JCVI, including additional disease quality of life losses, additional short-term disease phase costs, and quality of life, with the product ultimately funded [57]. In contrast, in Australia, a meningococcal vaccine product considered by the PBAC for funding saw the incorporation of productivity costs only considered in sensitivity analysis [58]. As of 2022, national funded coverage for meningococcal B is only for a small high-risk population in Australia [59].

#### 3.2.3. HTA Economic Evaluation of COVID-19 Vaccine Rollouts

As the pandemic has progressed, an increasing number of academic studies have estimated COVID-19 vaccine cost-effectiveness, employing healthcare system and societal perspectives, as well as hybrid approaches (Table 4). An evaluation of US-based COVID vaccination rollout strategies [60] took a US healthcare system perspective to inform rollout prioritisation, showing older populations (65 years^+^) were less costly and more effective than no vaccination. For the 50−64-year-old population, the vaccine incremental cost-effectiveness ratio (ICER) was USD 8000 per QALY; for 50–64-year olds with no serious medical conditions, and for 18−64-year olds with serious medical conditions, the ICER was USD 10,000 per QALY. For those aged 18 to 49 with no medical conditions, estimated ICER was USD 94,000, implying vaccines would not have been funded using current HTA practices in some jurisdictions [61,62]. 

In contrast, an economic impact analysis of the US [63] considered productivity costs and lost wage impacts when comparing vaccination to social restriction only scenario over a one-year time period. The inclusion of these indicated that the vaccine scenario saved society USD 19.8bn. A hybrid assessment combining HTA economic evaluation and economy-wide productivity impact assessment [64] used an age-structured dynamic transmission and economic model to evaluate different mass immunisation programmes in the UK. It considered economic evaluation outcomes from the traditional NICE healthcare system perspective [50], as well as a societal perspective including lost productivity impacts.

Compared with no vaccination, vaccination leads to positive incremental net monetary values in the best-case scenario ranging from GBP 12·0 to GBP 334.7bn; however, in the worst-case scenario, this ranges between–GBP 1·1 and GBP 56.9bn. This selection of modelled analyses confirms the substantial broader societal economic impacts caused by the pandemic globally.

#### 3.2.4. Promotion of HTA Evaluation Framework Revisions

In recent years, there have been some, albeit limited, formal efforts through government channels to promote a broader HTA evaluation framework. In 2016, the UK’s Cost-Effectiveness Methodology for Immunisation Programmes and Procurements (CEMIPP) group reviewed vaccine evaluation methodology following the JCVI Bexsero meningococcal vaccine decision. One recommendation proposed trialing of a broader scope of outcomes beyond health, including carer and family QALYs, non-family QALYs and non-health benefits [65]. In Australia, the 2021 Commonwealth Government *Parliamentary Inquiry into approval processes for new drugs and novel medical technologies in Australia* final report noted the restricted valuation scope of economic evaluations limited the ability of products with long-term health outcomes to demonstrate their true societal benefit. A broader societal evaluation perspective would not only be beneficial for vaccines, but for products with similar characteristics (e.g., treatments for rare diseases, cell and gene therapies and precision medicines) [66].

In recent times, professional societies, authors and groups have argued for new vaccine valuation methods. It has been noted that the UK’s JCVI is effectively undervaluing vaccines by not capturing spillover effects or externalities [67]. Further, it is considered COVID-19 has highlighted vaccines provide health, economic, and social benefits beyond that typically considered by HTA that should be taken into consideration [68]. UK commentary notes the considerable spending by the UK Government to support individuals, the economy and health services; if evaluated conventionally by NICE, these would not likely feature, drawing attention to need for broader HTA perspectives [69].

Building on work undertaken by The Professional Society for Health Economics and Outcomes Research (ISPOR) [70], Kamahl-Bahl et. al. [71] developed a valuation model of how the COVID-19 pandemic and its management highlights additional value elements (fear of contagion, insurance value and reduction in uncertainty, severity of disease, value of hope, real option value, scientific spill overs, equity, and family spillover effects which should be considered in vaccine valuation).

Similarly, Postma et al. [72] undertook a review of a previously developed vaccine valuation framework and concluded that, ‘gaps were evident for conventional societal perspective concepts (e.g., family/caregiver health and economic gains))’ and that ‘Few novel broader societal benefits were considered, and only in ad hoc cases.’ Looking forward, they noted ‘the top-three concepts for near-term consideration: macroeconomic gains (e.g., benefiting the economy, tourism), social equity and ethics (e.g., equal distribution of health outcomes, reduced health/financial equity gaps) and health systems strengthening, resilience and security (e.g., efficiency gains, reduced disruption, increased capacity).’

Consideration of appropriate valuation approaches has also been taken a step further with the argument it may be unclear if cost-effectiveness analysis is the best solution to finding appropriate vaccine prices. This position concludes the pandemic highlights that other measures of economic evaluation including budget impact, net health benefit, and net social benefit may be more appropriate for decision makers in selecting appropriately priced high-value solutions. When applied to pandemic situations, the perspective should include that of the whole society and economy [73].

In summary, literature review demonstrates existing HTA perspective and valuation frameworks provide for limited consideration of broader societal benefits. In this light, there appears to have been only limited formal efforts from governments and decision makers to consider development of broader formal HTA valuation frameworks for funding decision making. Post-COVID, international modelled economic evaluation studies have clearly demonstrated the broader societal impacts of COVID-19 and there have been continued efforts professionally and academically to develop potential broader valuation frameworks, with particular emphasis on broader societal effects capture and valuation deemed an increasing priority.

## 4. Discussion

Throughout the pandemic, Australian government policy has prioritised population health and prevention of healthcare system overwhelm. Prior to population-wide vaccination rollout, this was achieved through social and border restrictions. However, our modelling demonstrates this came at a significant cost to the Australian economy. Eventual restrictions removal and the opening up of society saw the Australian economy recover, with modelling showing material positive GDP and employment outcomes.

In particular, Australia’s education and tourism sectors, both reliant on the physical movement of international populations, were seriously impacted by the pandemic, with modelling showing their significant recovery. Sensitivity analysis reinforces this, demonstrating the material effect the timely availability of effective vaccines, vis-à-vis other countries, may have on the Australian economy.

Modelling shows rollout of vaccination from quarter one of 2021 seeing positive projected employment benefits (reflecting actual Australian employment outcomes) relative to the non-vaccination scenario. While the incremental benefit declines over time, it nonetheless remains at a significant level (greater than 100,000 jobs). This reflects recovery in conditions as work-from-home restrictions, school closures, and other physical distancing measures are lifted, naturally reversing the productivity and demand-driven losses assumed for 2020. It is relevant to note that employment levels, which were sharply depressed through the middle quarters of 2020, approached no COVID levels by the end of 2021.

The analysis also provides insight into longer term effects on the Australian economy. For example, Australia’s population declines via net migration under both scenarios, with an estimated 2.7% less than a no COVID scenario in quarter 2 2023, compared to 1.4% less with vaccination. The permanent reduction in Australia’s workforce across 30 months shows the positive effect of readily available vaccines on Australia’s long-term economic landscape.

The model employs a time horizon (30 months) much shorter than that typically used in HTA (potentially up to a lifetime, particularly for vaccines) [28]. Several points should be noted. Firstly, the CGE modelling aimed to estimate the incremental economic outcomes as the vaccination programme was rolled out. CGE modelling is a complex forecasting tool reliant on dozens of general assumptions and inputs regarding the operation of an economy. The modelling incorporated adjustments to capture the impact of the COVID ‘shock’ on the economy. At the time modelling simulations were computed (start of 2022), these shock assumptions were largely set, reflecting the understood COVID ‘decision problem’. That is, Australia had achieved the required levels of population-wide vaccination to enable removal of most restrictions and it was anticipated that the COVID pandemic would over time transition to a seasonal endemic virus that generally, would not involve ongoing restriction measures. The model also benefits from using actual economic data for a material proportion of the analysis period and as results demonstrate, the incremental economic impacts following rollout largely reach ‘steady state’ by early to mid-2023. In summary, this means the time horizon appropriately captures the decision problem reflecting the specified COVID shock, with analysis demonstrating the magnitude of economic impacts following a vaccination rollout.

In this regard, model results demonstrate the significant negative impacts on tourism and education sectors, which are reliant on physical movement of people. It is intuitive that without removal of restrictions, these sectors would see negative longer term economic impacts. There is also the potential that an opening up of Australia at a later date than that used in the model would still see relative economic losses, as consumers permanently re-orient their demand to other international markets. Further, as highlighted above, the pandemic is likely to have sufficient long-term effects on Australia’s economic capabilities (i.e., net migration).

It may be suggested that the actual economic activity after the onset of COVID compared to the economic situation immediately prior might yield an empirical measure of the impacts of COVID and of vaccination, and that this could be used for comparison purposes. However, the historical record through to quarter 2 of 2021 reflects a large number of factors (past investments, non-COVID related changes in world trading conditions, etc.) having no connection with the pandemic. For example, while a vaccination rollout ultimately facilitated people movement, it does not compel tourists and students to return to Australia; as economic agents (‘consumers’), they still have their own individual (and as a collective) decision making processes.

As such, economic outcomes are not solely attributable to vaccination and indeed reflect many influences including national and international government policies, prevailing economic trends, and resulting independent consumer behaviour. Indeed, as 2022 has unfolded, Australian and international economic outcomes have been subject to forces both domestic and international that have been only partly related to the pandemic, or alternatively, largely unrelated to the pandemic (e.g., global conflicts).

Nevertheless, the timely population-wide rollout of effective COVID vaccines was clearly an integral component in ‘unlocking’ society and opening the economy. Australia’s GDP performance since early 2021 tends to suggest that the economy has been on a path of recovery, with the ABS noting the contribution that domestic and international border opening has had [38], which in turn was predicated largely on Australia collectively reaching a specified population-wide vaccination level.

The pandemic evolved unpredictably, with continual shifts in the prevailing variant, health outcomes, healthcare system capacity and government policy responses. Economic modelling presented reflects ultimately prevailing pandemic circumstances, but it is possible circumstances could have unfolded differently (e.g., with different order of pandemic waves (Omicron and Delta), or more or less serious waves). However, analysis can largely be only based on the ultimate scenarios experienced. Further, the pandemic and the analysis crucially demonstrate the existence of the potential for infectious diseases to have uncertain, unpredictable and large-scale impacts on society.

Analysis, predicated on the exogenous shock of the pandemic, indicates the difference between the vaccination and no-vaccination scenarios steadies by early 2023. The pandemic has seen the Australian economy adapt over this time, with reallocation of resources and structural adjustments. The exact pathway after mid-2023 cannot be predicted with certainty. Potentially significant exogenous shocks could occur subsequently, however these cannot be forecast, and it is desirable that analysis assess the ‘state of play’ as it was best known. Ceteris paribus, in the future there may be a gradual convergence of economic outcomes under the ‘with’ and ‘without’ vaccination scenarios. Nonetheless, a material economic benefit of vaccination is expected to be evident for some period of time, including due to the underlying nature of the industries particularly affected and the cumulative long-term effect the pandemic has had on Australian economic capabilities (e.g., on net migration).

The pandemic has clearly demonstrated that infectious diseases and associated vaccines have unique characteristics with the potential to affect society at large, causing impacts beyond health and the healthcare system. Despite this, current HTA methodologies do not incorporate these broader value dimensions. In many senses, it is the capability for HTA valuation frameworks to capture the true value of impacts such uncertainty may generate that is of relevance.

The COVID-19 pandemic represents a historically unique situation, with the urgency and nature of government policy responses reflecting the speed, range, and depth of impacts. As such, it is acknowledged that practically, it would have been infeasible for vaccine procurement decisions to have occurred via standard HTA processes. Nonetheless, the VURM modelling analysis illustrates societal effects such as broader macroeconomic impacts clearly demonstrate the value of the timely availability of vaccines.

Reflecting the largely unpredicted original COVID-19 outbreak, there is therefore a continued risk that COVID in various forms may be present for years to come and in potentially equally serious (or worse) forms. Although society is transitioning to a ‘pandemic recovery’ phase, the dynamic nature of the virus means that having a valuation framework that provides for the uncertainty such virus waves may present is appropriate. 

Even for conditions of a smaller epidemiological scale, economic productivity impacts can be a particularly relevant issue where broader societal effects may influence the assessed value of an intervention. In part, this relates to how HTA decision makers regarding such broader societal impact evidence. At present, many HTA agencies, including Australia’s PBAC, only consider such analyses as ‘supplementary’ [28], with PBAC decisions, e.g., for vaccines, demonstrating they do not shape decision making [55].

With an increasing trend towards personalised medicines, cell and gene therapies expected in Australia [66], this may create challenges for timely reimbursement and availability of new cutting-edge therapies. For example, patients for who these treatments may be applicable (e.g., those with rare diseases) may face considerable, lifelong costs which fall outside of the traditional healthcare system and experience severely reduced quality of life, including employment and economic productivity, due to the debilitating nature of their conditions.

Since pandemic commencement, the Commonwealth Government has spent AUD 311bn on economic stimulus and AUD 31bn to support the healthcare system [6], an explicit acknowledgement of the need to address the pandemic’s impact on Australia’s society, economy, health, and wellbeing. Under current PBAC HTA Guidelines, such large-scale expenditures would not be considered in base case analysis [28]. In comparison, the costs of vaccination deployment, including vaccine procurement [74], and administration and distribution costs [75,76], estimated in total at AUD 12.6 billion, would appear to be a fraction of such support measures. 

The expected transition of COVID-19 to an endemic disease emphasises the rationale for taking a broader societal perspective for the HTA applied to vaccines. As literature review shows, there has been increasing international consideration of potential HTA approaches to valuation that provide for a broader scope.

The COVID-19 pandemic serves as a strong exemplar for a broader HTA perspective, with similar principles potentially applicable to other infectious diseases and other therapeutic areas. Failure to incorporate broader societal benefits risks undervaluing important medical innovations, limiting the utility of HTA as a timely decision-making instrument to facilitate investments in the Australian population’s health. With a future likely to increasingly feature innovative medicines, this risks the overall health outcomes of Australians.

While beyond the scope of this paper, there are issues worth noting that would need to be addressed to progress broader HTA valuation frameworks in practice. Firstly, there is a clear need to engage with key government and HTA agencies to progress policy development on this issue, as currently most efforts appear to be coming from industry organisations and academics. Secondly, there is a fundamental need to define both the scope of broader societal impacts (with consideration potentially of quantitative and qualitative factors), as well as how they would be identified, measured and valued individually. Thirdly, it is worth noting that HTA may require consideration of a range of approaches (novel models etc.). It seems that current HTA analysis approaches are too narrow to truly elucidate these types of economic impacts and value, with modelling software potentially being required to be adapted to be fit to capture broader elements of value. 

## 5. Conclusions

The COVID-19 pandemic saw many national governments implement social and border restrictions to mitigate population health and the healthcare system impacts. As societies experienced large-scale health and economic impacts, governments moved quickly to secure population-level vaccination stocks needed to address these effects and move society back to ‘normal’ operation. Economic modelling of restrictions removal in Australia demonstrates significant positive impacts for the Australian economy (GDP, key export industries, employment, and government finances). These benefits extend beyond health, healthcare system, and COVID-patient specific productivity benefits that might be directly attributed to a vaccination program and are orders of magnitude greater than the financial costs of vaccination purchase, distribution, and administration. While the economic recovery was the result of numerous factors, timely population wide rollout of effective COVID vaccines clearly played an integral role.

Our findings suggest that wider consideration of impacts is justified for HTA for vaccines and other health technologies, with the current scope of HTA valuation frameworks limited in their consideration of broader societal impacts. However, there are increasing efforts following the pandemic to construct such broader valuation frameworks and efforts should be made to consider how these can be incorporated into HTA decision making.

## Figures and Tables

**Table 1:** Summary of literature search approach.

Research Element	Description
Current international HTA evaluation framework perspectives	Summary of international HTA evaluation guidelines, including for vaccines.
International HTA evaluation framework considerations	Current or recent formal reviews of HTA evaluation frameworks.
International and Australian HTA funding decisions	Vaccine-specific HTA assessments where scope of value considered was material to evaluation outcomes.
COVID-19 vaccine rollout HTAs	Published international HTA-based economic evaluations of various COVID-19 vaccination rollout strategies. These are academic in nature and reflect the application of country-specific HTA frameworks, analyses that use broad economic evaluation principles and metrics (e.g., GDP), or potentially hybrid approaches. These are not formal HTAs used in government funding decision making, but rather modelled applications, that based on HTA principles and methodologies, demonstrate value implications.

Abbreviations: ‘COVID-19′, coronavirus; ‘HTA’, health technology assessment.

**Table 2:** Estimated impact on Commonwealth Government budget (AUD m, revenues less expenses).

Scenario	2019–2020	2020–2021	2021–2022	2022–2023
Open with vaccine (change from no COVID)	−AUD 92,717	−AUD 151,831	−AUD 63,458	−AUD 8481
Closed without vaccine (change from no COVID)	−AUD 92,717	−AUD 142,688	−AUD 215,737	−AUD 123,942
Difference between open and close scenario	−AUD 0	−AUD 9143	AUD 152,279	AUD 115,461

**Table 3:** Costs and outcomes included in the base case evaluation of vaccine interventions by international HTA agencies.

	Costs			Outcomes
	Healthcare	Societal		Societal
Country/HTA Body	Direct Healthcare Costs	Broader Government/Payer Costs	Broader Societal Costs	Direct Patient Health Outcomes
Australia (PBAC) ^1^	✓	S.A.	S.A.	✓
New Zealand (PHARMAC)	✓	×	×	✓
Germany (STIKO) ^2^	✓	S.A.	✓	✓
Sweden (FoHM) ^3^	✓	✓	✓	✓
UK (NICE/JCVI) ^4^	✓	S.A.	S.A.	✓
Canada (NACI) ^5^	✓	✓	✓	✓
The Netherlands (ZIN/CoV) ^6^	✓	✓	✓	✓
France (CTV/HAS) ^7^	✓	✓	S.A.	✓
Belgium (MoH) ^8^	✓	×	S.A.	✓
United States (ACIP) ^9^	✓	×	✓	✓

Abbreviations: ‘ACIP’, Advisory Committee on Immunization Practices; ‘CoV’, Committee on Vaccinations; ‘CTV’, Technical Vaccination Committee; ‘FoHM’, Public Health Agency of Sweden; ‘HAS’, Haute autorité de santé; ‘HTA’, health technology assessment; ‘JCVI’, Joint Committee on Vaccination and Immunisation; ‘KCE’, Healthcare Knowledge Centre; ‘NACI’, National Advisory Committee on Immunization; ‘PBAC’, Pharmaceutical Benefits Advisory Committee; ‘PHARMAC’, Pharmaceutical Management Agency; ‘S.A.’, sensitivity analysis/supplementary analysis; ‘STIKO’, Standing Committee on Vaccination; ‘ZIN’, Zorginstituut Nederland. Notes: ^1^ PBAC provides for costs/savings or socially relevant outcomes in domains such as education, housing or justice, or economic productivity impacts. Also, in circumstances where the beneficiaries of health or other relevant outcomes are broader than the treated patient population (e.g., community, carers, dependants). ^2^ STIKO guidance provides for productivity costs to patients and caregivers in uncertainty analysis. ^3^ Considers societal consequences including productivity loss of patients and caregivers. ^4^ The JCVI adopts the evaluation methods of NICE. The NICE perspective is that of the National Health Service (NHS) and personal social services (PSS), e.g., residential care and social worker costs. In exceptional circumstances when requested by the Department of Health and Social Care in the remit for the evaluation, the scope will list requirements for adopting a broader perspective on costs. ^5^ Canada’s NACI Guidelines for reporting model-based economic evaluations of vaccination programs in Canada guidance on perspective notes, ‘publicly funded health system and societal at minimum, with societal considerations inclusive of, ‘productivity losses, consumption, and costs and outcomes of non-health sectors’. ^6^ In the Netherlands, the Committee on Vaccinations (under the Health Council) advises the Minister of Health, Welfare and Sport about public vaccination programs. It has 7 assessment criteria for inclusion of a vaccine in a public programme, one of which is economic. Although it does not specify perspective, ZIN, responsible for economic evaluation in the Netherlands to determine health insurance coverage, uses a societal perspective, which can include patient and family costs (e.g., costs of informal care) and productivity costs. ^7^ Although not required, the CTV can commission a full economic analysis. The CTV engages with the HAS. For the purposes of comparison, HAS HTA Guidelines have been assumed. The relevant HTA advisory body, National Authority for HTA (Comité Economique des Produits de Santé) makes recommendations on clinical evidence only. ^8^ In Belgium, the NITAG is located within the Ministry of Health. HTA evaluation in Belgium adopts the payer perspective. ^9^ The US’ ACIP provides for all relevant medical costs regardless of payer, time costs of patients in seeking and receiving care, time costs of informal caregivers, transportation costs, effects on future productivity and consumption, and other effects occurring outside the healthcare sector.

**Table 4:** Selection of HTA studies evaluating cost-effectiveness of COVID-19 vaccination strategies.

Paper Name and Authors	Health or Societal Perspective	Publication Date
The potential public health and economic value of a hypothetical COVID-19 vaccine in the United States: Use of cost-effectiveness modelling to inform vaccination prioritisation [60]	Health	6 January 2021
Economic value of vaccine to address the COVID-19 pandemic: a U.S. cost-effectiveness and budget impact analysis [63]	Health	31 August 2021
The potential health and economic value of SARS-CoV-2 vaccination alongside physical distancing in the UK: a transmission model-based future scenario analysis and economic evaluation [64]	Health and Societal	9 August 2021

## Data Availability

The economic modelling was undertaken using a large-scale Computable General Equilibrium model known as the Victorian University Regional Model (VURM). VURM is run using the GEMPACK software. VURM is a large model with a large database. For each of the simulations reported in this paper, a zip archive is available which covers all inputs to the modelling process. These archives are created specially by the GEMPACK software and can be used to recreate exactly the simulations conducted. They are freely available for anybody with access to GEMPACK and can be obtained by contacting Philip Adams–philip.adams@vu.edu.au.

## Appendix A. CGE Modelling Employed to Estimate Economic Impacts of COVID-19 and Vaccination Rollout

### Appendix A.1. Introduction

In this paper, we use a modified version of the VURM [19] to understand the impacts of COVID-19 vaccination on the Australian economy. Our objective is to answer the following question: How has the development of a vaccine that allowed COVID-19 restrictions to be relaxed in 2021 impacted the Australian economy?

### Appendix A.2. Analysis Scenarios

Our analysis is based on the modelling of three scenarios, starting in the first quarter of 2020 and ending in the second quarter of 2023: (1) A hypothetical no COVID scenario in which COVID-19 does not exist and the Australian economy grows in line with pre-COVID trends; [two with COVID scenarios] (2) Opening with vaccination, reflecting history through to the second quarter of 2021 and most-probable estimates of a fairly smooth recovery arc thereafter based on current knowledge of the effectiveness of vaccination; and (3) Closed without vaccination, reflecting history through to the second quarter of 2021 and then our best guess of what happens if vaccines prove ineffectual, leading to little or no opening up before the second half of 2023.

Comparing the Opening with vaccination scenario to the no COVID scenario gives our best estimates of the effects of COVID with effective vaccination. Comparing the Closed without vaccination scenario to no COVID yields our best estimates of the effects of COVID with prolonged lockdowns relative to a world without COVID. Comparing Opening with Closed yields an estimate of the economic consequences of effective vaccination in the current COVID-19 setting for Australia.

Note that at the time of running the simulations, statistics through to and including the second quarter of 2021 were available for a range of key economic variables from the Australian Bureau of Statistics (ABS), including national accounts, balance of payments and labour force statistics. These data have been incorporated into our scenarios.

### Appendix A.3. The VURM

#### Appendix A.3.1. VURM Introduced

The VURM is a detailed, dynamic, 77-industry CGE model which covers all Australian states and territories and major economic agents [29]. CGE modeling is an established macroeconomic technique commonly used by government agencies to inform policy discussions and decisions [30]. It uses actual economic data to estimate how economic activity might react to changes in policy, technology, or other external factors (‘shocks’). The modelling aims to show the difference between two alternative future economic states, i.e., ‘with’ and ‘without’ the proposed change, by considering interactions between sections of the economy [31].

For each region, a representative household, all levels of government (local, state and federal), international trade (i.e., imports and exports) are captured. Supply and demand for each regionally produced commodity reflects optimising behaviour. Regional industries are assumed to use intermediate inputs, labour, capital and land in a cost-minimising way, while operating in competitive markets. Households seek to purchase bundles of utility-maximising goods and services. Regions are linked via interregional trade, interregional migration and capital movements, and governments operate within a fiscal federal framework [29,31].

Investment in each regional industry is of expected rates of return and is a function of local and foreign investment. Capital creators assemble physical capital for each regional industry.

To parameterise VURM, CoPS relies on data from various sources, including ABS Census data, Agricultural Census data, state accounts data, and international trade data. The core VURM model database underwent a significant update during the first half of 2020 to incorporate the ABS 2016/17 Input-Output data release [77], together with updated Government Financial Statistics data from ABS cat. No. 5512.0 [78].

In solving VURM, we undertake two parallel model runs: a base case simulation and a scenario simulation. The base case simulation is a business-as-usual forecast for the period of interest. The scenario simulation is identical to the base case simulation in all respects, other than the addition of shocks describing the scenario under investigation. We report results as cumulative deviations (either percentage or absolute) away from base case in the levels of variables in each period of the scenario simulation.

VURM-generated results for a particular year flow on into the database, where the form the basis for analysis of the subsequent year (e.g., capital stocks, debt, savings). The model is solved with the GEMPACK software package [79].

#### Appendix A.3.2. Modelling Simulation Design

The two COVID scenarios are generated as deviations away from the hypothetical no COVID scenario. The no COVID projection contains business-as-usual assumptions for productivity and other key economic drivers, and makes no allowance for COVID-19, vaccination and containment policies.

The COVID scenarios deviate from no COVID in response to seven sets of shocks:

1. Productivity shocks relating to physical distancing, etc.,
2. Physical distancing (demand) shocks,
3. Shocks to export demand (from COVID and COVID containment in the Rest of the World),
4. Fiscal shocks,
5. Shocks to population due to reduced net overseas migration;
6. Labour productivity shocks due to working days lost from testing through to mortality; and
7. Shocks to health care demand associated with testing and patient care.

Each set of shocks has a magnitude and path to recovery that is based on a combination of evidence, legislation and judgement based on assumed recovery paths.

#### Appendix A.3.3. Productivity Shocks

The physical distancing strategy for containment of COVID-19 required people to work from home where possible, resulting in productivity losses. For workplaces with staff on-site, productivity is also negatively impacted, due to COVID-specific measures required to be implemented.

Estimates of resulting productivity losses of these measures ranging from 1 per cent and 3 per cent, equivalent to losing 5–15 min of productive time per 8-h workday, were initially assumed.

For each industry, the productivity loss is put in place for 2020Q2 and unwound in quarter 1 of 2021, by which time we assume that strict hygiene measures are relaxed, and to the extent to which they remain, businesses have adapted. The extent of productivity loss and recovery are the same in both COVID scenarios.

#### Appendix A.3.4. Domestic Containment Measures

Domestic containment shocks relate to limits on social gatherings and non-essential travel of the type primarily experienced, for example, in Victoria during the second half of 2020. To represent domestic containment, negative shocks are applied to domestic final demand in selected industries.

Shocks to demand were devised using the most detailed data on industry production (20 ANZSIC classes) provided in the ABS’ quarterly national accounts publication. Changes in production were allocated across demand reflecting shares in the existing VURM database. For the Opening with vaccination Scenario shocks were applied in line with observed data through to and including 2021Q2, and progressively unwound through to quarter 1 of 2022. Thereafter, it is assumed that domestic containment measures cease.

For the Closed without vaccination scenario, we assumed that demand remained suppressed through to the end of the projection period in line with suppression levels existing in quarter 1 of 2021 (when vaccinations became available).

#### Appendix A.3.5. International Shocks

For both COVID scenarios, shocks to exports of non-service commodities (everything except tourism and education) in 2020 are derived from an average of the actual impacts on growth by country, weighted according to Australia’s export profile to each country. The average impact on the growth of our trading partners is weighted for each set of commodities. We adjust to reflect the fact that in a COVID scenario, country activity is likely to be more inward looking Such adjustments are expected to be pronounced for agriculture and manufacturing, as Australian exports are relatively expensive and will be impacted by reducing spending in trading partner jurisdictions.

After 2020, export recovery is governed by the nature of the scenarios to be modelled. In the Opening with vaccination scenario, we assume export changes implied by actual data to quarter 2 of 2021. Thereafter, smooth recovery is assumed, such that by quarter 1 of 2022 there is no change from assumed no COVID levels. For the Closed scenario we assume the deviations in export demand in quarter 4 of 2020 continue through to the end of the projection period.

Shocks to travel-related exports, including air transport, accommodation and dining are substantial.

#### Appendix A.3.6. Fiscal Shocks

In response to the COVID pandemic, the Commonwealth government initially announced three support packages to the value of AUD 150 billion, while the Reserve Bank of Australia put in place an AUD 90 billion funding facility to assisted banks with their lending practices to business [80]. The first support package of AUD 17.6 billion was announced on the 12th of March 2020 and provided support for business investment and cash flow, with additional targeted support for the most severely affected sectors, regions and communities, and direct payments to lower income households [81]. The second rescue package, worth AUD 66.1 billion [82], included further support to households and assistance to business. JobKeeper, provides payment to workers totaling approximately AUD 70 billion [83].

JobKeeper was to be paid over the 2nd and 3rd quarters of 2020. Subsequently, the Government extended the scheme (at a reduced rate) until the end of the 1st quarter of 2021 [84]. JobKeeper is represented by wage subsidies and transfer payments. Wage subsidies reduce the employer labour costs, while transfer payments (from government to households) do not change incentives.

#### Appendix A.3.7. Net Overseas Migration and Australia’s Population

The pandemic has seen international movement dramatically decline. Border closure policies thus impact this population growth.

#### Appendix A.3.8. Labour Productivity Shocks Due to Working Days Lost

The containment policies of the pandemic have led to significant job losses. It has also led to a substantial number of working days lost for those employed as a result of time spent being tested and in isolation and recovery Those lost days represent a reduction in labour productivity—hours worked per week. The loss of labour productivity because of these factors will be higher in the Opened with vaccination scenario than in the Closed without vaccination scenario because the prevalence of COVID is higher in the former than the later. Key assumptions and sources reflecting these calculations include:

- Considers cases in the working age population (i.e., 15 to 64);
- Assumed average 38 weekly work hours in accordance with Fair Work Commission [85];
- Reflects historical and projected instances of testing, isolation, and positive COVID-19 cases (including stay at home, hospitalisation, ICU and death);
- Historical instances are sources from www.covidlive.com.au [4], which in turn are sourced from Commonwealth and respective state and territory government sources. Projections are based on the published work of the Grattan Institute [86];
- Days of lost work reflects government-mandated protocols, testing procedures, observed testing response times as they evolved over the course of the pandemic. For example, testing evolved from PCR tests to increasing use of rapid antigen tests (RATs), with consequential evolution in testing-based isolation times and government guidance on isolation for a non-hospitalised case evolving from 14 days to 7 days;
- In absence of recent published data, the COVID vaccination scenario assumes same COVID virus virulency as most recent strain (Delta) prior to modelling development applies to all subsequent analysis from December 2021 quarter. This means health complications are of the same severity (i.e., duration);
- Adjustments are made to reflect the working week, i.e., applying an apportionment factor of 5/7ths to reflect weekends;
- Time lost for hospitalisation reflects data published by the Australian Institute of Health and Welfare (AIHW) in September 2021 and reflects the period January to June 2020 [87];
- Conservative estimation of total time off work, i.e., does not assume additional time (e.g., post-hospitalisation home recuperation, additional granted time off work, ‘long COVID’ effects, complications etc.).

#### Appendix A.3.9. Shocks to Health Care Expenditure Associated with Testing and Patient Care

The pandemic increases the demand for health services. This increase is exogenously imposed in the VURM model using information on expenditures include spending on vaccination, testing facilities and additional support to public hospitals required to cater for severe COVID cases. COVID-related healthcare expenditures assumed reflect published Commonwealth and state and territorial budget statements for the period 2019–2020 to 2022–2023.

## Appendix B. CGE Modelling Results

Appendix B provides a detailed summary of CGE modelling results by key macroeconomic indicators (GDP, real tourism exports, real education exports, population growth, employment and Commonwealth Government budget), for each scenario, by quarter, between January 2020 and June 2023.

### Appendix B.1. Real GDP

**Table A1:** Percentage points (%) change in GDP with and without vaccine vs. no COVID scenario.

Scenario	2020(1)	2020(2)	2020(3)	2020(4)	2021(1)	2021(2)	2021(3)	2021(4)	2022(1)	2022(2)	2022(3)	2022(4)	2023(1)	2023(2)
Opening with vaccine	−1.2	−8.5	−5.9	−3.5	−2.2	−2.0	−3.0	−2.6	−2.1	−1.7	−1.5	−1.3	−1.3	−1.3
Opening with vaccine (upper scenario)	−1.2	−8.5	−5.9	−3.5	−2.2	−2.0	−3.0	−2.6	−2.1	−1.4	−1.0	−0.7	−0.7	−0.7
Opening with vaccine (lower scenario)	−1.2	−8.5	−5.9	−3.5	−2.2	−2.0	−3.0	−2.6	−2.1	−2.0	−2.0	−1.8	−1.9	−1.9
Closed without vaccine	−1.2	−8.5	−5.9	−3.5	−7.8	−7.2	−6.2	−5.3	−4.8	−4.7	−4.9	−5.0	−5.0	−5.1

**Table A2:** AUD bn change in Australian GDP with and without vaccine vs. no COVID scenario.

Scenario	2020(1)	2020(2)	2020(3)	2020(4)	2021(1)	2021(2)	2021(3)	2021(4)	2022(1)	2022(2)	2022(3)	2022(4)	2023(1)	2023(2)
Opening with vaccine	−AUD 9.9	−AUD 49.3	−AUD 34.4	−AUD 20.3	−AUD 13.0	−AUD 11.9	−AUD 16.0	−AUD 13.1	−AUD 10.4	−AUD 8.6	−AUD 7.6	−AUD 6.5	−AUD 6.6	−AUD 6.7
Opening with vaccine (upper)	−AUD 9.9	−AUD 49.3	−AUD 34.4	−AUD 20.3	−AUD 13.0	−AUD 11.9	−AUD 16.0	−AUD 13.1	−AUD 10.4	−AUD 7.1	−AUD 5.2	−AUD 3.5	−AUD 3.5	−AUD 3.4
Opening with vaccine (lower)	−AUD 9.9	−AUD 49.3	−AUD 34.4	−AUD 20.3	−AUD 13.0	−AUD 11.9	−AUD 16.0	−AUD 13.1	−AUD 10.4	−AUD 10.1	−AUD 10.0	−AUD 9.4	−AUD 9.7	−AUD 10.0
Closed without vaccine	−AUD 9.9	−AUD 49.3	−AUD 34.4	−AUD 20.3	−AUD 38.2	−AUD 35.4	−AUD 30.8	−AUD 26.2	−AUD 24.2	−AUD 23.9	−AUD 24.7	−AUD 25.5	−AUD 26.0	−AUD 26.5
Difference (with vs. without)	AUD 0.0	AUD 0.0	AUD 0.0	AUD 0.0	AUD 25.2	AUD 23.5	AUD 14.8	AUD 13.1	AUD 13.8	AUD 15.3	AUD 17.1	AUD 19.0	AUD 19.4	AUD 19.8

### Appendix B.2. Real Tourism Exports

**Table A3:** Percentage (%) reduction in real tourism exports with and without vaccine vs. no COVID scenario.

Scenario	2020(1)	2020(2)	2020(3)	2020(4)	2021(1)	2021(2)	2021(3)	2021(4)	2022(1)	2022(2)	2022(3)	2022(4)	2023(1)	2023(2)
Opening with vaccine	−25.5	−92.0	−92.0	−90.0	−85.0	−80.0	−75.0	−65.0	−52.0	−28.0	−15.0	−10.0	−10.0	−10.0
Opening with vaccine (upper)	−25.5	−92.0	−92.0	−90.0	−85.0	−80.0	−75.0	−65.0	−52.0	−9.8	−1.0	0.0	0.0	0.0
Opening with vaccine (lower)	−25.5	−92.0	−92.0	−90.0	−85.0	−80.0	−75.0	−65.0	−52.0	−36.0	−27.0	−24.0	−24.0	−24.0
Closed without vaccine	−25.5	−92.0	−92.0	−90.0	−87.0	−85.0	−85.0	−85.0	−85.0	−85.0	−85.0	−85.0	−85.0	−85.0

### Appendix B.3. Real Education Exports

**Table A4:** Percentage (%) reduction in real education exports with and without vaccine vs. no COVID scenario.

Scenario	2020(1)	2020(2)	2020(3)	2020(4)	2021(1)	2021(2)	2021(3)	2021(4)	2022(1)	2022(2)	2022(3)	2022(4)	2023(1)	2023(2)
With vaccine	−20.0	−30.0	−82.0	−81.0	−77.0	−74.0	−68.0	−65.0	−55.0	−30.0	−20.0	−15.0	−15.0	−15.0
With vaccine best	−20.0	−30.0	−82.0	−81.0	−77.0	−74.0	−68.0	−65.0	−55.0	−15.5	−1.5	0.0	0.0	0.0
With vaccine worst	−20.0	−30.0	−82.0	−81.0	−77.0	−74.0	−68.0	−65.0	−55.0	−42.0	−35.0	−32.0	−32.0	−32.0
Without vaccine	−20.0	−30.0	−82.0	−81.0	−76.0	−76.0	−76.0	−76.0	−76.0	−76.0	−76.0	−76.0	−76.0	−76.0

### Appendix B.4. Population Growth

**Table A5:** Percentage (%) points reduction in population growth with and without vaccine vs. no COVID scenario.

Scenario	2020(1)	2020(2)	2020(3)	2020(4)	2021(1)	2021(2)	2021(3)	2021(4)	2022(1)	2022(2)	2022(3)	2022(4)	2023(1)	2023(2)
With vaccine	0.00	−0.08	−0.30	−0.53	−0.75	−0.93	−1.07	−1.18	−1.27	−1.34	−1.38	−1.40	−1.40	−1.40
With vaccine best	0.00	−0.08	−0.30	−0.53	−0.75	−0.93	−1.07	−1.18	−1.27	−1.37	−1.47	−1.57	−1.67	−1.77
With vaccine worst	0.00	−0.08	−0.50	−0.70	−1.00	−1.30	−1.60	−1.80	−2.00	−2.10	−2.20	−2.40	−2.58	−2.74
Without vaccine	0.00	0.00	0.20	0.17	0.25	0.37	0.53	0.62	0.73	0.76	0.82	1.00	1.18	1.34

### Appendix B.5. Employment

**Table A6:** Reduction in national headcount employment (000s) with and without vaccine vs. no COVID scenario.

Scenario	2020(1)	2020(2)	2020(3)	2020(4)	2021(1)	2021(2)	2021(3)	2021(4)	2022(1)	2022(2)	2022(3)	2022(4)	2023(1)	2023(2)
With vaccine	−13	−640	−418	−205	−228	−210	−207	−204	−200	−197	−194	−191	−188	−186
With vaccine best	−13	−640	−418	−205	−228	−210	−207	−204	−200	−178	−170	−163	−155	−148
With vaccine worst	−13	−640	−418	−205	−228	−210	−207	−204	−200	−217	−218	−220	−222	−223
Without vaccine	−13	−640	−418	−205	−352	−397	−382	−367	−353	−340	−327	−314	−302	−291

### Appendix B.6. Commonwealth Government Budget

**Table A7:** Impacts on Commonwealth Govt budget (Financial year, AUD m changes)—Revenue—with and without vaccine vs. no COVID scenario.

Scenario	2019−2020	2020−2021	2021−2022	2022−2223
With vaccine	−AUD 18,731	−AUD 26,634	AUD 5460	AUD 2839
Without vaccine	−AUD 18,731	−AUD 38,770	−AUD 56,997	−AUD 22,113
Difference (with vs. without)	AUD 0	AUD 12,136	AUD 62,457	AUD 24,952

**Table A8:** Impacts on the Commonwealth Govt budget (Financial year, AUD m changes)—Expenses—with and without vaccine vs. no COVID scenario.

Scenario	2019−2020	2020−2021	2021−2022	2022−2023
With vaccine	AUD 73,986	AUD 125,197	AUD 68,918	AUD 11,320
Without vaccine	AUD 73,986	AUD 103,918	AUD 158,739	AUD 101,829
Difference (with vs. without)	AUD 0	AUD 21,279	−AUD 89,822	−AUD 90,508

## Appendix C. Literature Search Methods Summary

Pragmatic scoping literature searches were conducted for the following topics:

- Targeted pragmatic literature searches for post-pandemic HTA-based COVID-19 vaccine evaluation studies,
- Efforts to reform HTA valuation frameworks and
- Options proposed to enhance HTA vaccine valuation scope were also conducted.

Searches of the databases PubMed and Scopus were conducted originally in late October 2021, with subsequent search again in March to May 2022. Key search terms included:

HTA Guidelines Review
For each shortlisted country search terms combinations included:
  - UK: ‘UK’ ‘NICE’ ‘methods’ ‘JCVI’ ‘HTA’ ‘vaccines’
  - Australia: ‘Australia’ ‘PBAC’ ‘guidelines’ ‘vaccines’
  - US: ‘US’ ‘ACIP’ ‘economic’ ‘evaluation’ ‘vaccines’
  - New Zealand: ‘NZ’ ‘PHARMAC’ ‘PFPA’ ‘vaccines’
  - France: ‘France’ ‘HAS’ ‘CTV’ ‘economic’ ‘evaluation’ ‘vaccines’
  - The Netherlands: ‘Netherlands’ ‘NL’ ‘vaccination committee’ ‘economic’ ‘evaluation’ ‘vaccines’
  - Canada: ‘Canada’ ‘NACI’ ‘vaccines’ ‘economic’ ‘evaluation’
  - Germany: ‘Germany’ ‘STIKO’ ‘economic’ ‘evaluation’ ‘vaccines’
  - Belgium: ‘Belgium’ ‘KCE’ ‘economic’ ‘evaluation’ ‘guidelines’ ‘vaccines’
  - Sweden: ‘Sweden’ ‘economic’ ‘evaluation’ ‘guidelines’ ‘vaccines’
HTA COVID-19 evaluations:
  - *‘COVID-19′*
  - *‘HTA’*
  - *‘Cost-effectiveness’*
  - *‘Vaccine’*
  - *‘CUA’*
  - *‘Cost per QALY’*
HTA valuation framework reform:
  - ‘HTA’ ’guideline’ ‘COVID’ ‘value’ ‘vaccines’ ‘reform’ ‘perspective’ ‘societal’
